# Lateral Membrane Heterogeneity Regulates Viral-Induced Membrane Fusion during HIV Entry

**DOI:** 10.3390/ijms19051483

**Published:** 2018-05-16

**Authors:** Rodion J. Molotkovsky, Veronika V. Alexandrova, Timur R. Galimzyanov, Irene Jiménez-Munguía, Konstantin V. Pavlov, Oleg V. Batishchev, Sergey A. Akimov

**Affiliations:** 1 Laboratory of Bioelectrochemistry, A.N. Frumkin Institute of Physical Chemistry and Electrochemistry, Russian Academy of Sciences, 31/4 Leninskiy Prospekt, 119071 Moscow, Russia; swinka87@gmail.com (R.J.M.); timur.galimzyanov@gmail.com (T.R.G.); olegbati@gmail.com (O.V.B.); 2 Faculty of Physics, M.V. Lomonosov Moscow State University, 1-2 Leninskie Gory, 119991 Moscow, Russia; supercrazybird@gmail.com; 3 Department of Theoretical Physics and Quantum Technologies, National University of Science and Technology “MISiS”, 4 Leninskiy Prospekt, 119049 Moscow, Russia; 4 Department of Engineering of Technological Equipment, National University of Science and Technology “MISiS”, 4 Leninskiy Prospekt, 119049 Moscow, Russia; w0r3ss@gmail.com; 5 Laboratory of Electrophysiology, Federal Clinical Center of Physical-Chemical Medicine of FMBA, 1a Malaya Pirogovskaya Street, 119435 Moscow, Russia; qpavlov@mail.ru; 6 Department of Physics of Living Systems, Moscow Institute of Physics and Technology (State University), 9 Institutskiy Lane, Dolgoprudniy, 141700 Moscow Region, Russia

**Keywords:** membrane fusion, human immune-deficiency virus, fusion peptide, raft, theory of elasticity

## Abstract

Sphingomyelin- and cholesterol- enriched membrane domains, commonly referred to as “rafts” play a crucial role in a large number of intra- and intercellular processes. Recent experiments suggest that not only the volumetric inhomogeneity of lipid distribution in rafts, but also the arrangement of the 1D boundary between the raft and the surrounding membrane is important for the membrane-associated processes. The reason is that the boundary preferentially recruits different peptides, such as HIV (human immunodeficiency virus) fusion peptide. In the present work, we report a theoretical investigation of mechanisms of influence of the raft boundary arrangement upon virus-induced membrane fusion. We theoretically predict that the raft boundary can act as an attractor for viral fusion peptides, which preferentially distribute into the vicinity of the boundary, playing the role of ‘line active components’ of the membrane (‘linactants’). We have calculated the height of the fusion energy barrier and demonstrated that, in the case of fusion between HIV membrane and the target cell, presence of the raft boundary in the vicinity of the fusion site facilitates fusion. The results we obtained can be further generalized to be applicable to other enveloped viruses.

## 1. Introduction

Cellular membranes are multicomponent, and interaction between the components of a membrane results in its lateral inhomogeneity and formation of liquid ordered domains, often referred to as “rafts” [1]. Rafts are thicker than the surrounding membrane [2] and participate in various processes, including cell signaling and regulation of the membrane-associated protein activity [3,4]. The main role of rafts in these processes is believed to be related to formation of specific lipid environment for transmembrane and peripheral proteins, enabling the protein function. Some experimental data indicate raft involvement in the process of reception of different viruses on the surface of cellular membranes [5]. In the works [6,7], it has been demonstrated that raft boundaries preferentially recruit fusion peptides such as the gp41 of HIV. Remarkably, lack of preferential recruitment of influenza virus hemagglutinin fusion peptide to the raft boundary was pointed out in these publications. Besides, in the case of HIV fusion efficiency proved to be enhanced when raft boundary was in the vicinity of the fusion site.

Raft is thicker than the membrane around it [2]; deformations of the lipid matrix tend to compensate the hydrophobic mismatch and minimize the area of contact of raft lipid hydrocarbon chains with water occurring near its boundary [8]. Earlier, it was shown in a series of theoretical papers [9,10,11,12] that the minimum of the elastic energy of membranes corresponds to a nonzero shift of domain boundaries in opposing monolayers of the membrane. The calculated equilibrium magnitude of the shift is too small (~2–3 nm) to be optically detected. However, such a shift was observed in different conditions in several works by means of molecular dynamics modeling [13,14,15]. In particular, in [15], raft boundary shift was detected in bilayer with calcium-induced phase separation in a membrane consisting of charged and neutral lipids. Presence of such a transient zone near the raft boundary can act as an attractor for line-active components with spontaneous curvature [11], since their incorporation into the domain boundary also allows minimization of the deformation energy. Similarly, the preferential distribution of HIV fusion peptides into the raft boundary observed in the works [6,7] can be a consequence of relaxation of the membrane elastic energy caused by such incorporation of the proteins.

**Figure 1:**
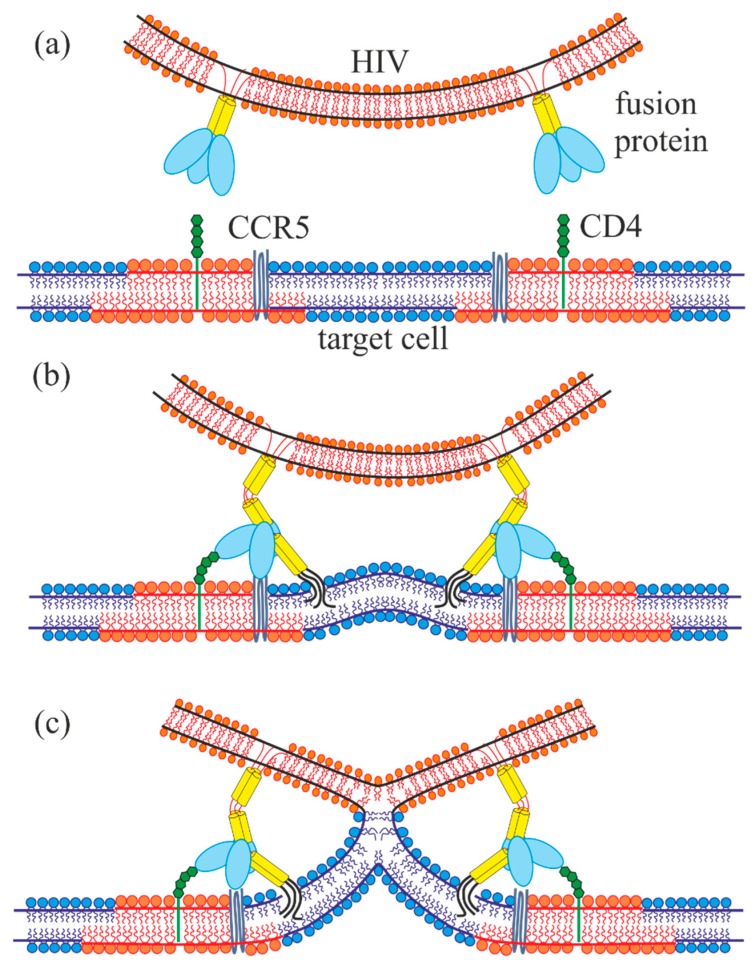
Schematic representation of the initial stage of T-cell infection by HIV. (**a**) Viral membrane in the vicinity of the target cell membrane; the target cell membrane consists of lipids of raft (highlighted in red) and non-raft (shown in blue) phases. (**b**) Interaction of fusion proteins with CD4 receptors (green) and CCR5 co-receptors (midnight blue) on the cell surface accompanied by conformational transitions of fusion proteins and incorporation of fusion peptides (shown in black) into the cellular membrane. (**c**) Formation of stalk—a structure, in which the contact monolayers of membranes already fused, while the distal ones have not yet.

Earlier, we have demonstrated that there is a strong correlation between the depth of the viral fusion peptide incorporation into a monolayer of the target cell membrane (which is equivalent to generation of the membrane curvature) and the height of the energy barrier for virus-induced membrane fusion [16,17]. According to the generally adopted theory, membrane fusion occurs in several stages [18]. At the first stage, the contacting monolayers of membranes fuse, resulting in formation of a stalk—the structure, in which the contacting monolayers already merged, while the distal monolayers have not. The stalk then expands, the distal monolayers come into contact, and the so-called fusion diaphragm is formed. After that, a pore is formed in the fusion diaphragm, thus completing the fusion process. Formation of a stalk requires overcoming an energy barrier associated with the membrane topological rearrangement. According to the number of theoretical estimates [19,20,21], this barrier is about several tens of *k_B_T* (*k_B_T* ~ 4 × 10^−21^ J). It strongly depends on the distance between the fusing membranes: the smaller the distance, the lower the barrier. Fusion efficiency is determined by the height of the energy barrier (the lower the barrier, the more effective fusion is). In the process of cell infection by viruses, the barrier is partly compensated by the action of specific proteins known as “fusion proteins” [22,23], which undergo conformational transition aimed to bring the viral and the target cell membranes in a closer juxtaposition. In the case of HIV, the rearrangement is triggered by interaction of subunits of the fusion protein with CD4 receptors and CCR5 co-receptors on the surface of the target cell membrane [24,25]. Such an interaction results in exposure of the hydrophobic fusion peptide with its subsequent incorporation into the target cell membrane. In the present work, we attempt to elucidate how interaction of fusion peptides with raft boundaries can affect efficiency of virus induced fusion. Our calculations are based on the liquid crystal elasticity theory adapted to lipid membranes (detailed description of the methodology is available in the works [26,27]). In the framework of this theory, the membrane is treated as a continuous liquid crystal medium subjected to elastic deformations. The deformations are caused by fusion peptides incorporated into the membrane, along with the hydrophobic thickness mismatch at the raft boundary. The elastic parameters of the membrane as a whole take into account, in particular, the specific interactions of lipids with each other and with the proteins embedded in the membrane. We assume that CD4 receptors nucleate rafts around them, and the boundaries of the rafts in the opposing monolayers of the cell membrane are misaligned (shifted relative to each other) (see Figure 1a), according to the results obtained in [9]. HIV fusion peptides incorporate deeply into the target cell membrane, inducing negative curvature in the membrane [28]. Due to such mode of incorporation, the peptides are capable of changing the energy barriers associated with the membrane topological rearrangement, inclusively those related to stalk formation. In our calculations, we assume that the membrane fusion occurs at the expense of cooperative action of several HIV fusion proteins (see Figure 1b,c). Presently, there is limited understanding of this issue in the available publications [24,29]. Some authors suggest that one or two trimers participate in the fusion, whereas others claim that several trimers are needed [30,31]. We also take into account the evaluations carried out in the work [20], according to which the total work done by proteins should be of the order of 100 *k_B_T*. At the same time, the estimate of the energy liberated in the process of the conformational transition of one fusion protein trimer amounts to several tens of *k_B_T* [18]. These estimates also indirectly indicate the cooperative effect of several proteins in the process of fusion. Together with the axial symmetry of the fusion stalk structure, that leaves us with few peptides, located around some center-stalk. That picture justifies the assumption of the cooperative action of several fusion peptides leading to the ring-like insertion into the membrane. The assumption of cooperative action of several fusion peptides allow greatly simplifying the calculations by means of considering cylindrically symmetrical ring of fusion peptides, known as fusion rosette [32].

In the course of fusion, the proteins must continuously withstand pulling forces directed to pull them out of the membranes, both viral and target. For many viruses, lipid composition of the membranes is similar to that of rafts of plasma membrane, i.e., in particular, they are enriched by cholesterol [33,34,35,36]. Fusion proteins have transmembrane domains anchored in the viral membrane [37], often comprising specific cholesterol-recognizing amino acid consensuses, allowing strong binding to cholesterol [38]—and, consequently, to the viral membrane. Strong binding to the target membrane is provided by the high hydrophobicity of fusion peptides or hydrophobic structures performing analogous functions. Simple estimation of the difference of single fusion peptide energies in the water and in the membrane yields Δ*W_h_* = 2*πR*_1*fp*_*Lσ*_0_ ≈ 60–80 *k_B_T*, where *R*_1*fp*_ ~ 1 nm is the characteristic diameter of the fusion peptide; *L* ~ 2 nm is the characteristic length of the fusion peptide; *σ*_0_ ~ 40–50 mN/m is the surface tension on water/hydrophobic peptide interface. Thus, according to our calculations presented below, the total elastic stress developed in fusing membranes is hardly sufficient to pull a single fusion peptide out of the target membrane.

## 2. Results

### 2.1. Dependence of the Equilibrium Position of the Fusion Peptide Upon Incorporation Depth

**Figure 2:**
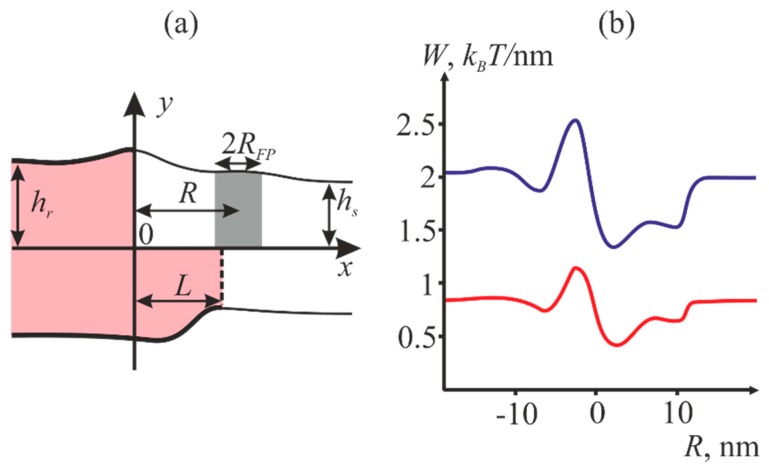
(**a**) Schematic representation of the model of a peptide incorporated into the membrane with the raft. The raft area is shaded in pink and outlined by bold lines, the surrounding membrane is shown in white and outlined by thin lines. *L* designates the shift of the monolayer raft boundaries. The area occupied by fusion peptides is shown in gray. The width of the fusion peptide is 2*R_FP_*. The distance from the center of fusion peptide to the center of coordinates is designated as *R*. (**b**) Dependence of the equilibrium energy of the membrane *W* (expressed as the energy per unit length of the raft boundary) upon the coordinate *R* of the fusion peptide. The blue curve corresponds to the case of the half-width *R_FP_* equal to 1 nm, the red one—to the case of the half-width *R_FP_* equal to 0.5 nm. The equilibrium thicknesses of the raft monolayer and the monolayer of the surrounding membrane are designated as *h_r_* and *h_s_*, respectively.

In order to define the preferred location of the fusion peptide with respect to the raft boundary, consider a membrane containing a liquid ordered domain with the peptides incorporated into it to a certain depth. The fusion peptide is considered to represent a membrane inclusion of a given geometry characterized by a spontaneous curvature. We assumed the system to have translational symmetry, i.e., to be quasi-unidimensional. As we demonstrated earlier, the characteristic profiles of the energy of a membrane with peptide inclusions of finite size are qualitatively identical to the energy profiles of unidimensional inclusions incorporated into the membrane to the same depth [39]. We use a Cartesian coordinate system with the origin at the boundary of the bilayer raft (see Figure 2a). The axes are selected so that the *Oy* axis is perpendicular to the membrane surface, whereas the *Ox* axis is perpendicular to the raft boundary and lies in the membrane plane. The relative shift of the raft boundaries is designated as *L*. Incorporation of fusion peptides into the monolayer induces additional deformations of the bilayer, which is accounted for by means of introducing appropriate boundary conditions. The peptide is assumed to be parallel to the raft boundary. We vary the location of the center of fusion peptide with respect to the raft boundary and calculate the equilibrium energy of the corresponding deformations of the membrane. The complete width of the fusion peptide, 2*R_FP_*, is assumed to be smaller than the shift *L* of the monolayer raft boundaries. The dependence of the membrane equilibrium energy on the position *R* of the peptide with respect to the center of coordinates is shown in Figure 2b.

In our calculations, we assumed the elasticity moduli typical for the majority of lipid membranes, namely: *B* = 10 *k_B_T*, *K* = 10 *k_B_T*/nm^2^ [40,41] for the monolayer splay and tilt moduli, respectively. The surface tension *σ* of a monolayer is assumed at 0.01 *k_B_T*/nm^2^. The raft monolayer thickness *h_r_* was taken equal to 2 nm; the surrounding membrane monolayer thickness *h_s_* was 1.5 nm [2]. The only difference of the raft from the surrounding membrane was in the equilibrium thickness. The equilibrium width *L* of transient zone between the domain boundaries in the two juxtaposed monolayers of the membrane were also found by means of minimization of the membrane deformation energy, and equaled 2 nm.

Figure 2b illustrates that the fusion proteins preferentially partition into the liquid disordered part of the membrane in the immediate vicinity of the raft boundary.

### 2.2. Dependence of the Stalk Formation Energy Barrier on the Presence of a Raft

**Figure 3:**
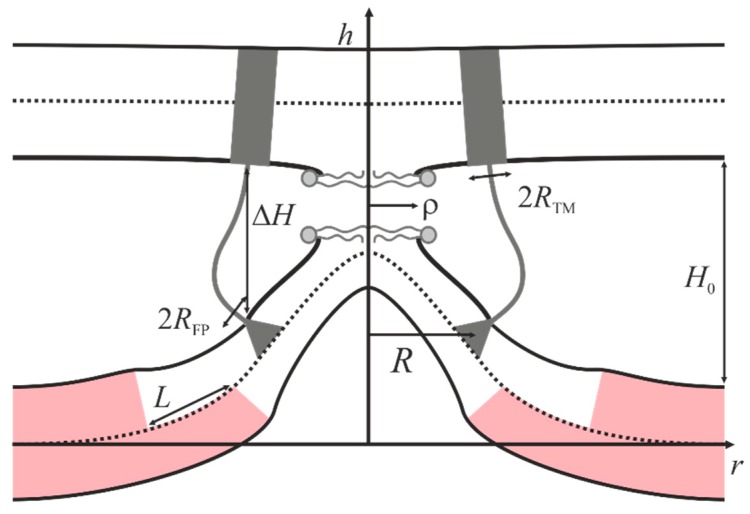
Schematic representation of the model. Distance Δ*H* between fusion peptides and transmembrane domains of proteins in membranes is used as the reaction coordinate. Transmembrane domains (half-width of *R_TM_*) are schematically shown by gray rectangles, fusion peptides (half-width of *R_FP_*) are shown by gray triangles. *H*_0_ is the equilibrium distance between the membranes, ρ is the radius of the hydrophobic face formed in the area of maximal proximity of the membranes. Raft area is highlighted in pink. The viral membrane is shown on the top, and the cellular membrane is shown at the bottom.

We use the data on preferential redistribution of lipids described in the previous section to calculate the stalk formation energy barrier in the presence of a raft in the target cell membrane using the example of deep incorporation of the fusion peptide, as is the case of HIV. We assume that in the non-disturbed conditions at a sufficiently large distance from the fusion site the membranes are planar, since their curvature radius (~50 nm in the case of virus) greatly exceed the size of the fusion site (~10 nm) and the characteristic distance of deformation decay (~1 nm). The transmembrane domains and fusion peptides of HIV are modeled by two coaxial annuli of the radius *R* with the half-widths of *R_TM_* and *R_FP_*, respectively. The transmembrane domains are modeled as annular inclusions penetrating the entire depth of the viral membrane bilayer, whereas the fusion peptides—as annular inclusions incorporated into one monolayer of the target membrane to relatively large depth (see Figure 3).

We consider a cylindrical coordinate system *Ohr*, and put its origin *O* to the surface of monolayer interface of the lower membrane. The radial axis *Or* is assumed to lie in the plane of the monolayer interface, and *Oh* axis—to be directed along the axis of rotational symmetry of the system. This system is virtually unidimensional, as its properties vary only along the radial coordinate *r*. We assume that the fusion proteins bring the membranes to a certain distance *H*_0_, at which the hydration repulsion forces [42] equilibrate the attraction force applied by the proteins. It is also assumed that, in the course of fusion, the distance Δ*H* between the annulus of transmembrane domains and the annulus of fusion proteins in the target cell gets smaller, whereas the distance between the membranes in the area remote from the fusion rosette remains unchanged and equal to *H*_0_ (see Figure 3). Due to the large size of fusion proteins and their transmembrane (TM) domains as well as their high density in the viral membrane, we assume that the deviation of transmembrane domains from the initial equilibrium position can be neglected. An energy barrier associated with the hydration repulsion forces has to be crossed in order to bring the membranes closer [43]. In the conditions when fusion peptides tend to decrease the distance between the membranes, the hydration repulsion of the bilayers presumably results in a lateral displacement of the lipid polar heads from the membrane contact area [20]. Thus, hydrophobic defects are formed in the contact monolayers of the bilayers undergoing fusion [44]; the radius of the hydrophobic defect is designated as *ρ* in Figure 3. Such defects can act as nucleation centers for monolayer fusion, since their formation results in local disordering of the hydration layers and occurrence of hydrophobic attraction between the contact monolayers [45], ultimately resulting in stalk formation.

In the course of analysis, all the elastic parameters of the membranes were assumed to be the same as in the previous section. Besides that, the following values taken from the work [46] were used for the hydration repulsion parameters: wedging pressure *P*_0_ = 60 *k_B_T*/nm^3^, hydration interaction characteristic length *ξ_h_* = 0.35 nm. The hydrophobic attraction characteristic length *ξ_f_* was assumed equal to 1 nm in compliance with the experimental data reported in [45]. The half-width of the transmembrane domain *R_TM_*, as well as the half-width of the fusion peptide *R_FP_* were assumed equal to 1 nm.

**Figure 4:**
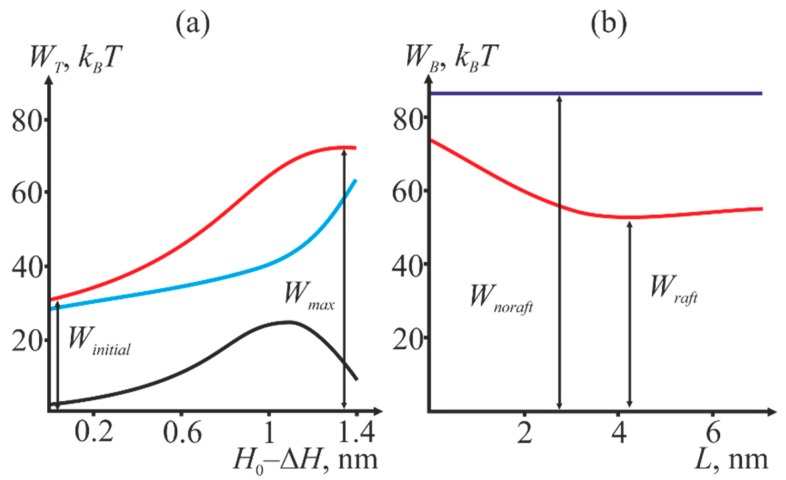
(**a**) Dependence of the system total energy *W_T_* (shown in red) on the reaction coordinate *H*_0_ − Δ*H*. The blue curve is the deformation energy of the two membranes, the black one is the energy of hydration repulsion of the membranes and hydrophobic interaction of the defects. The graph corresponds to the values of *R* = 3 nm, *L* = 3 nm, *H*_0_ = 3 nm. (**b**) Dependence of the energy barrier *W_B_* to stalk formation on the width *L* of the raft transient zone (shown in red). The energy barrier in the absence of raft in the target cell membrane is shown in blue. The graph corresponds to the values of *R* = 3 nm, *H*_0_ = 4 nm.

We calculated the dependence of the system total energy *W_T_* on the reaction coordinate *H*_0_ − Δ*H*. The hydrophobic patch radius was variable. An example of the dependence of total energy on the reaction coordinate is shown in Figure 4a. The dependencies allow calculating the energy barrier to the stalk formation. The barrier height *W_B_* is calculated as a difference between the maximal energy on the trajectory and the initial energy:

*W_B_* = *W_max_* − *W_initial_* (1)

The initial energy *W_initial_* was not equal to zero since membrane deformations caused by incorporation of fusion peptides and compensation of the hydrophobic mismatch at the raft boundary are already factored into it. Then, we varied the length *L* of the raft transient zone and calculated the energy barrier for each value of *L* at fixed radius *R* of the protein annulus and distance *H*_0_ between the membranes. Figure 4b illustrates an example of the dependence of the barrier height upon *L*.

For each fixed *H*_0_, the energy barrier is minimized against the transient zone width *L*. This corresponds to the minimum of the red curve in Figure 4b (designated as *W_raft_*). Then, we compare the obtained value with the barrier height in the absence of the raft (designated as *W_noraft_*). Two values: the minimal height of the barrier *W_raft_* and difference Δ*W* are of crucial importance for our analysis. The latter is defined as

Δ*W* = *W_noraft_* − *W_raft_* (2)

**Figure 5:**
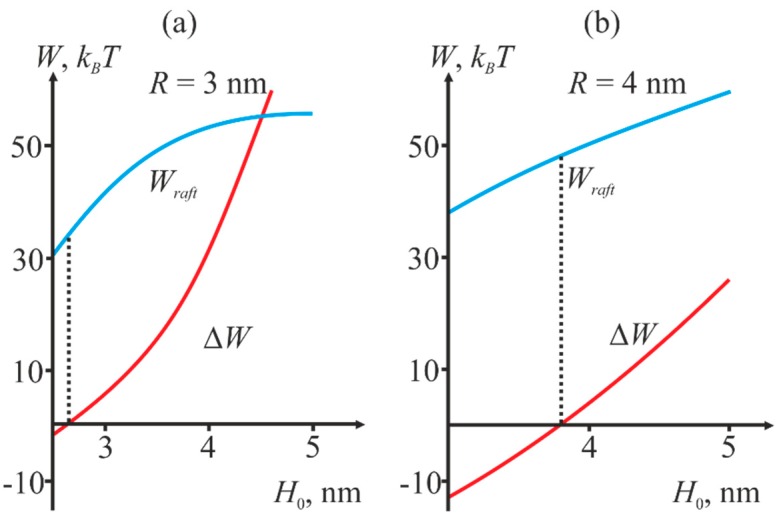
Dependence of *W_raft_* (blue curve) and Δ*W* (red curve) on the initial distance *H*_0_ between the membranes at different values of the fusion rosette radius *R*. (**a**): *R* = 3 nm; (**b**) *R* = 4 nm. The height of the barrier corresponding to intersection of the red line with the abscissa corresponds to the minimal energy needed to achieve the stalk state in the presence of a raft (shown by vertical dashed lines).

If Δ*W* is positive, the presence of the raft near the fusion site decreases the energy barrier, i.e., facilitates fusion, otherwise the raft presence hinders it. Figure 5 illustrates the dependence of *W_raft_* and Δ*W* on the initial distance *H*_0_ between the membranes at different values of the fusion rosette radius *R*.

Thus, we obtain that depending on the initial distance *H*_0_ between the membranes, presence of a raft boundary near the fusion site can decrease the energy barrier to stalk formation, thus facilitating fusion. At small values of *H*_0_, the energy is lower in the absence of rafts, and vice versa.

## 3. Discussion

As of today, many authors admit that the presence of rafts is a prerequisite for successful reception of HIV. Rafts are believed to be necessary for normal functioning of CD4 receptor and CCR5 co-receptor [47,48,49,50]. Recent experiments indicate that rafts interact with the HIV fusion peptides and facilitate fusion of the viral membrane and target cell membrane [6,7]. The largest effect is achieved when HIV is in the vicinity of the phase separation boundary between the raft and the surrounding membrane. The authors qualitatively explain this finding considering the elastic properties of the membrane in the neighborhood of the raft boundary. In particular, influence of the membrane deformation in the vicinity of the raft boundary on incorporation of the fusion proteins and the membrane curvature near the fusion site is discussed. Analysis of our model revealed that the membrane deformation energy is minimal when the peptide incorporates into the non-raft phase in the vicinity of the raft boundary (Figure 2b). It means that the HIV fusion peptide has to partition into the neighborhood of the raft boundary; and the deeper the peptide incorporates into the membrane the higher the partitioning is. This explains the selectivity of the results obtained in the work [6]: both in the case of HIV and influenza virus, the fusion peptides are preferentially recruited into the raft boundary. However, in the case of influenza virus, the energy minimum is relatively shallow and is readily crossed at the expense of thermal fluctuations. This result, along with the results obtained in the work [11], suggests the following hypothesis: raft boundaries constitute the zone of preferential recruitment of any peptide with non-zero spontaneous curvature.

We have shown that depending on the initial distance *H*_0_ between the membranes, presence of the raft boundary can either decrease or increase the energy barrier to stalk formation (Figure 5). Decrease of *H*_0_ value results in reduction of the energy barrier *W_raft_*, and simultaneously with that presence of the raft boundary becomes less favorable for fusion (Δ*W* becomes negative). The equilibrium distance *H*_0_ between the membranes is determined by the balance of hydration repulsion forces and the attraction induced by fusion peptides, and it can vary depending on the number of proteins in the fusion rosette and on the curvature of the membranes undergoing fusion. According to various estimates [17,20,51], this value is of the order of several nanometers. According to Figure 5, small *H*_0_ values correspond to the situation when rafts are not favorable for fusion, while the large values—to the situation when rafts facilitate it. The critical height of the barrier, at which rafts still favor fusion, decreases with the increasing radius *R* of the rosette and amounts to 45 *k_B_T* at *R* = 4 nm and 35 *k_B_T* at *R* = 3 nm. These values are in excellent agreement with the theoretical estimates obtained earlier [20,21]. Thus, we have obtained that, in the case of fusion between HIV and target cell membranes raft boundary in the vicinity of the fusion site can facilitate fusion. As can be readily seen from a typical curve shown in Figure 4b, asymmetry of the raft boundary provides additional relaxation of the deformation energy and results in decrease of the barrier by several *k_B_T*.

In this paper, we theoretically show that in the case of HIV, insertion of viral fusion peptide into the region of the raft boundary promotes viral fusion. In our work, we use a mechanistic approach for the membrane description successfully used for theoretical investigation of various membrane systems [8,9,11,16,17]. In the framework of this approach, the insertion of any peptide into the membrane is described by the averaged geometric characteristics: depth and width of the insertion. Thus, peptide insertion disturbs the ordered arrangement of lipids in the membrane and induces curvature within it, which is shown experimentally and theoretically in a number of works [52,53,54]. This induction of curvature has a key effect on a variety of intracellular processes, including membrane fusion [55,56]. Due to the averaging of geometric parameters determining the curvature, this mechanism does not depend on the specific protein or peptide and can be generalized to all those cases in which the curvature is induced. Some experimental evidence suggests that the interaction of inclusions of fusion peptides or hydrophobic structures performing analogous functions (e.g., hydrophobic loop in the case of Semliki forest virus) with lipid rafts is not an exclusive feature of HIV, but is rather characteristic for other enveloped viruses, e.g., Semliki Forest virus [57] and Ebola virus [58]. This implies that the results we obtained can be generalized to describe the processes of fusion of different viruses.

## 4. Materials and Methods

### 4.1. Energy of the Membranes with Peptide Inclusions

The lipid membrane with a peptide inclusion and a domain of liquid ordered phase (raft) is investigated. The membrane deforms because of the raft being thicker than the surrounding membrane, and because of incorporation of fusion peptides into it. Membrane deformation is treated in the framework of Hamm and Kozlov model [41]. In order to describe monolayer deformations, a field of unit vectors of directors, **n**, characterizing the average orientation of lipid molecules, is introduced. The field of directors is defined on a certain surface within the monolayer. The shape of the surface is determined by the unit vectors **N** normal to it (directed towards the inter-monolayer surface of the membrane). We take into account two deformation modes—tilt and splay. The deformations are attributed to the so-called neutral surface, where the splay and lateral extension deformations are independent on each other. According to the experimental data obtained in [59], the neutral surface lies in the transient area between the lipid polar heads and hydrophobic chains at the depth of ~0.5 nm from the external surface of the lipid monolayer. Splay deformation is qualitatively described by divergence of the director along the neutral surface, whereas the tilt deformation is described by the tilt vector field **t** = **n**/(**nN**) − **N** ≈ **n** − **N**. We assume the membrane deformation is small, and hence the energy of deformed monolayer counted from the state of planar monolayer can be expressed as [41]

(3) $$W=\int \left(\frac{B}{2}{\left(\mathrm{div}n\right)}^{2}+\frac{K}{2}t^{2}+\sigma \right)dS-\sigma A_{0}$$

**Figure 6:**
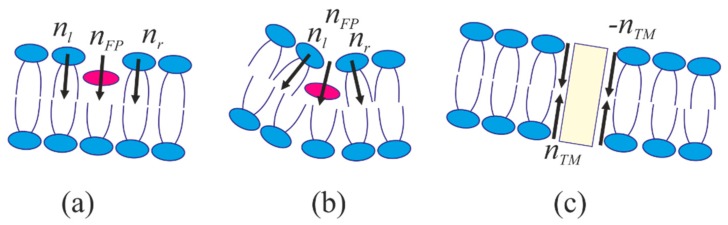
Schematic representation of fusion peptide domains (shown as magenta ellipse) in a bilayer. (**a**) intermediate incorporation depth; (**b**) deep incorporation; (**c**) transmembrane domain (shown as yellow rectangle). Black arrows show the boundary director orientations.

Here, *B* and *K* are splay and tilt moduli, respectively, *σ* is the lateral tension of the monolayer; *dS* is the neutral surface area element; *A*_0_ is the area of the neutral surface in the initial undisturbed state. We introduce Cartesian system of coordinates with the origin at the right-hand side boundary of the bilayer raft (see Figure 2a). The axes are selected so that the *Oy* axis is perpendicular to the membrane surface, whereas the *Ox* axis is perpendicular to the raft boundary and lies in the membrane plane. Smallness of deformations implies that the director projection upon the *Ox* axis is much smaller than unity. All the functions defining the membrane shape and its deformations depend on only one spatial coordinate in the unidimensional case. The vector fields of directors, normals to the neutral surface, and tilts can be replaced with their projections on the *Ox* axis: **n** → *n_x_* = *n*, **N** → *N_x_* = *N*, **t** → *t_x_* = *t*. Director divergence transforms into its component directed along the *Ox* axis: div(**n**) → *dn*/*dx.* In addition to that, we only take credit for local volumetric incompressibility condition [41]:

(4) $$\Delta h=h_{0}-\frac{h_{0}{}^{2}}{2}{\left(\mathrm{div}n\right)}^{2}$$

where Δ*h* is the monolayer local thickness; *h*_0_ is the undisturbed monolayer thickness. The thickness of undisturbed raft is designated as *h_r_*, and differs from the thickness of undisturbed monolayer of the surrounding membrane, designated as *h_s_*. The location of the intermonolayer surface *m*(*x*) is defined as the distance from the *Oxy* plane to the intermonolayer surface measured in the given point *x* along a perpendicular to the plane *Oxy*. We define the location of the neutral surface of the upper monolayer *h_a_*(*x*) and of the lower monolayer *h_b_*(*x*) in a similar manner. Equation (4) along with the definitions of tilt vector (**t** = **n** − **N**), monolayer thickness (Δ*h_a_* = *h_a_*(*x*) − *m*(*x*), Δ*h_b_* = *m*(*x*) − *h_b_*(*x*)), and normal to the neutral surface of a monolayer (**N_a_** = **grad**(*h_a_*(*x*)), **N_b_** = −**grad**(*h_b_*(*x*))), relate the tilt angles *t_a_*(*x*) and *t_b_*(*x*) in the upper and lower monolayers with directors, *a*(*x*) and *b*(*x*) in these monolayers, as well as position of the intermonolayer surface, *m*(*x*). Thus, local incompressibility condition applied to two monolayers of the membrane decreases the number of independent functions characterizing the state of a membrane segment from five (*a*(*x*), *b*(*x*), *h_a_*(*x*), *h_b_*(*x*), *m*(*x*)) to three (*a*(*x*), *b*(*x*), *m*(*x*)). These three functions are sufficient to rewrite the elastic energy functional, Equation (3). In order to find the functional extremals, we vary it with respect to independent functions *a*(*x*), *b*(*x*), *m*(*x*) and obtain a system of three differential Euler–Lagrange equations, whose solutions are then substituted into the elastic energy functional, Equation (3). The expressions for functions *a*(*x*), *b*(*x*), *m*(*x*), obtained by solving the system of Euler–Lagrange equations contain free coefficients, which are determined by minimizing the energy with specified boundary conditions. The boundary conditions are dependent on the geometry of incorporation of fusion proteins and hydrophobic thickness mismatch at the raft boundaries. See more details of the methodology used for calculating the elastic energy in the works [8,9,60]. Incorporation of fusion peptide into the membrane is accompanied by lateral shift of the adjacent lipid molecules, and in general case—by tilt of the lipid molecules at the boundary to a certain angle with respect to the neutral surface of undeformed membrane. We designate the projection of director at the inner boundary of fusion protein layer (*r* = *R* − *R_FP_*) as *n_l_*, at the outer boundary (*r* = *R* + *R_FP_*)—as *n_r_* (Figure 6a,b). Besides that, fusion peptide can rotate in the membrane as a whole. To account for that, we designate the projection of the director describing this rotation as *n_FP_*. Obviously, *n_FP_* = (*n_l_* + *n_r_*)/2, i.e., *n_FP_* is an average of the directors at the inner and outer boundaries of the fusion peptide layer.

With the aid of geometric interpretation of the director, the difference between the directors at the inner and outer boundaries of the fusion protein layer (director discontinuity) can be expressed through the width of the annulus, 2*R_FP_*, and monolayer thickness, *h*_0_, as

(5) $$\begin{array}{l} a\left(R+R_{FP}\right)-a\left(R-R_{FP}\right)=0,\\ a\left(R+0\right)-a\left(R-0\right)=\frac{2R_{FP}}{\sqrt{h_{0}{}^{2}+R_{FP}{}^{2}}} \end{array}$$

for the cases of intermediate depth of insertion (corresponding to influenza virus) and deep insertion (corresponding to HIV) of fusion peptides, respectively (Figure 6a,b). Since fusion peptides concentrate in the vicinity of raft boundary, the monolayer thickness *h*_0_ in Equation (5) is assumed equal to half sum of the monolayer thicknesses of the raft and the surrounding membrane

(6) $$h_{0}=\frac{h_{r}+h_{s}}{2}$$

Incorporation of the fusion peptide into the contact monolayer of the target membrane to intermediate depth leads to rupture of the neutral surface. As the peptides rotate as a whole, the energy penalty of exposure of its hydrophobic parts to the water drives corresponding relative shift of the monolayer neutral surfaces at the inner and outer boundaries of the fusion peptide ring

*h_a_*(*R* + *R_FP_*) − *h_a_*(*R* − *R_FP_*) = 2*R_FP_n_FP_* (7)

In the case of deep incorporation of the peptide into contact monolayer, it is assumed that there is no discontinuity of its neutral surface, and thus

*h_a_*(*R* + 0) − *h_a_*(*R* − 0) = 0 (8)

### 4.2. Stalk Energy

The total energy of the stalk can be decomposed to contributions from the elastic deformations *W_e_*, from the hydration repulsion between the hydrophilic surfaces of the contact monolayers, *W_h_*, and from the attraction of the hydrophobic patches formed in the opposing membranes, *W_f_*:

*W*_T_ = *W_e_* + *W_h_* + *W_f_* (9)

We introduce cylindrical system of coordinates *Ohr*, with the origin *O* and axis *Or* in the plane of inter-monolayer surface of the bottom membrane, and the *Oh* axes coinciding with the rotation symmetry axis of the system (see Figure 3). Due to cylindrical symmetry, the system is effectively unidimensional, the fusion peptides and transmembrane domains of fusion peptides are circumferentially located, and all the parameters depend on the coordinate *r* only. As before, the membrane deformations are defined by the boundary conditions. In addition to the boundary conditions on the annulus of fusion peptides, we take into account the conditions imposed by the ring of transmembrane domains in the virus membrane and by the hydrophobic defect in the center of the fusion site.

The viral membrane deforms when transmembrane domains of fusion proteins incline with respect to its normal, leading to the entrainment of adjacent lipids. Deviation of the lipid orientation results in non-zero projection of the boundary director onto the *Or* axis. We designate its value as *n_TM_* (Figure 6c). Besides that, tilt of the transmembrane domains within the fusion rosette causes relative displacement of the neutral surfaces of monolayers on the inner (*r* = *R* − *R_TM_*) and outer (*r* = *R* + *R_TM_*) boundaries of the ring. Thus, we have the following boundary conditions

*a*(*R* ± *R_TM_*) = −*n_TM_*, *b*(*R* ± *R_TM_*) = *n_TM_*, *h_a_*_,*b*_(*R* + *R_TM_*) − *h_a_*_,*b*_(*R* + *R_TM_*) = −2*n_TM_R_TM_* (10)

In the area of tight membrane contact, lipid polar heads in both membranes can fluctuate laterally forming a round hydrophobic spot with the radius of *ρ*. Such a displacement occurs at the expense of membrane deformation, and effectively reduces hydration repulsion energy. Lateral displacement of polar heads causes the projections of the director onto the *Or* axis at the boundary of the hydrophobic spot to change as

(11) $$a\left(\rho \right)=-\frac{\rho }{h_{0}}$$

This condition satisfies two limiting cases, for which the values of the boundary director are known. Specifically, from the considerations of symmetry it is clear that in the case of zero radius hydrophobic defect (*ρ* = 0), the director at its boundary has to be zero. Besides that, when the radius of the defect equals the monolayer thickness (*ρ* = *h*_0_), lipid molecules are horizontally oriented, i.e., the director must be equal to −1. Besides fixing the director at the boundary of the hydrophobic spot, we also restrain the separation Δ*H* of the neutral surfaces of the contact monolayers of fusing membranes along the central circles of the protein rings, at *r* = *R*. Additionally, the distance between these neutral surfaces is maintained equal to *H*_0_ far from the fusion site, at *r* → ∞.

We utilize Israelachvili theory to express the interaction energy of two circular hydrophobic patches formed in the contact monolayers of viral and target membranes [45]

(12) $$W_{f}=2\sigma_{0}\pi \rho^{2}\left(1-\exp\left(-\frac{l}{\xi_{f}}\right)\right)$$

where *ξ_f_* is the characteristic length of hydrophobic interactions in water [45]; *l* is the distance between the hydrophobic spots; *σ*_0_ is the surface tension of the macroscopic phase separation boundary (water/lipid carbohydrate chains). The hydration repulsion energy is calculated according to [42,43] as

(13) $$W_{h}=P_{0}\xi_{h}\int \exp\left[-\frac{z\left(r\right)}{\xi_{h}}\right]dS$$

In this equation, *z*(*r*) is the separation of the membranes at the given coordinate *r*; *P*_0_ is the disjoining (or wedging) pressure, corresponding to maximal possible repulsion of contacting hydrophilic surfaces; *ξ_h_* is the characteristic length of decay of hydration repulsion. The integration in the Equation (13) is performed over the hydrophilic surface of the contact monolayers. In order to evaluate the integral in Equation (13), we use Derjaguin approximation [61], according to which the integration in the Equation (13) can be limited to the area in which the distance between the membranes changes by the value of *ξ_h_*, having replaced the deformed hydrophilic surfaces of contact monolayers with horizontal planes. If there are no hydrophilic surfaces in the membranes, integration in Equation (13) starts from *r* = 0. In case there are hydrophobic spots in the membranes, integration is performed from *r* = *r* + *L_h_*, making allowance for smearing of the boundary between the hydrophobic spot and the bulk membrane. Such smearing is caused by several factors—fluctuations of polar heads of lipids (the characteristic size of a polar head is ~0.8 nm), finite characteristic length of decay of the order parameter of the hydrophobic and hydrophilic interaction (~0.35 nm and 1 nm, respectively). We selected the value of *L_h_* ~ 1 nm.

## Abbreviations

HIV	human immunodeficiency virus
TM	transmembrane (domain)
